# Tribal casinos in California: the last vestige of indoor smoking

**DOI:** 10.1186/1471-2458-12-144

**Published:** 2012-02-25

**Authors:** David S Timberlake, Jun Wu, Wael K Al-Delaimy

**Affiliations:** 1 Program in Public Health, College of Health Sciences, University of California, Irvine, Anteater Instruction & Research Building, Irvine, CA, USA 92697-3957; 2 Department of Family and Preventive Medicine, University of California, San Diego, La Jolla, CA, USA

**Keywords:** Secondhand smoke, California tribal casinos, Smoking ban, Smoking prevalence

## Abstract

**Background:**

High levels of airborne particles from secondhand smoke have been reported in California Indian casinos. Yet, little is known regarding the smoking status of casino patrons, their avoidance of secondhand smoke while visiting, and their views on a hypothetical smoking ban.

**Methods:**

Predictors of visiting an Indian casino were assessed among participants of the 2008 California Tobacco Survey (n = 10, 397). Exposure to and avoidance of secondhand smoke were subsequently analyzed among a subset of participants who had visited a casino in the year prior to the survey (n = 3, 361).

**Results:**

Ethnic minorities, older individuals, current smokers and residents of sparsely populated regions of California were more likely than other demographic groups to visit a tribal casino. Avoidance of secondhand smoke was more frequent among the never smokers than former and current smokers, particularly those who last visited a casino lacking physical separation between non-smoking and smoking sections. The never smokers versus current smokers disproportionately expressed a willingness to extend their stay and visit again if smoking were prohibited.

**Conclusions:**

If casinos became smoke free, then it is anticipated that they would be visited by a significantly larger number of Californians, including both patrons and those who otherwise would not have visited a casino.

## Background

The state of California has long been regarded as a pioneer in the tobacco control movement in the United States. It was the first to develop a comprehensive tobacco control program in 1988 [1] and the first to enact a smoke-free workplace law in 1994 (i.e. Assembly Bill 13). The latter occurred in the wake of dozens of smoke-free restaurant ordinances that were passed in local communities throughout the state [2]. The smoke-free laws in California were opposed by the tobacco industry and its sponsored organizations (e.g., Beverly Hills Restaurant Association) who argued that such laws would cause economic loss for bars and restaurants in California [3]. However, no such long-term economic loss occurred following enactment of the indoor smoking ban in California [4]. As a likely consequence of California's commitment to tobacco control, the State's smoking prevalence has been less than the overall smoking prevalence in the U.S. for many years (e.g., 15.2% vs. 20.9%, respectively, in 2005 [5]). Outside of California, diminished revenue from smoke-free policies was also a concern for the gambling industry [6] due to the co-occurrence of gambling and cigarette smoking [7,8]. The tobacco industry collaborated with the gambling industry in financing economic studies, ventilation projects and lobbying activities against the smoke-free policies [9]. Loss of gaming revenue from enactment of smoke-free laws was reported in Victoria, Australia [10], but not in the U.S. states of Massachusetts [11] and Delaware [12].

Passage of the 1988 Indian Gaming Regulatory Act, based on the sovereignty of federally recognized Indian reservations, led to the establishment of numerous Indian casinos throughout California. Sovereignty also enabled Indian tribes to permit smoking in casinos despite passage of California's Assembly Bill 13. Aside from loopholes in the law allowing smoking in certain indoor settings (e.g., banquet facilities), Indian casinos represent the last vestige of indoor smoking where Californians are exposed to hazardous secondhand smoke. One recent study, which measured airborne fine particles in 36 of 58 California casinos [13], reported considerable variability in fine particle concentrations by level of separation between the non-smoking and smoking areas; for the casinos that had complete physical separation, fine particle levels were comparable to levels measured in the outdoor samples.

No study in the academic literature, to our knowledge, has assessed perceptions of secondhand smoke among casino patrons, a likely function of the difficulty in obtaining a representative sample. Analysis of one such representative sample from the 2008 California Tobacco Survey (CTS) is a unique opportunity to research secondhand smoke among patrons of tribal casinos in California. This also has international implications because of the provisions set forth in the World Health Organization's Framework Convention on Tobacco Control (FCTC). Article 8 of the Convention declares that public places and workplaces be free of secondhand smoke [14]. Yet, even in the countries that have ratified the FCTC, non-smokers continue to be exposed to secondhand smoke in public indoor settings (e.g., in Santiago, Chile [15]). Many countries have enacted a weak smoke-free policy that reflects the tobacco industry's "Courtesy of Choice" Program [16,17]. This program supports the designation of smoking and non-smoking sections in indoor public settings, similar to what is observed in the California tribal casinos. An examination of patrons' perceptions of secondhand smoke in the casinos, therefore, may be informative for policymakers as well as casino and hospitality industries in California and abroad.

Using data from the 2008 California Tobacco Survey, we aimed to assess smoking prevalence by casino visitation, predictors of casino visitation, avoidance of secondhand smoke among casino patrons, and willingness to extend one's stay and visit again if smoking were prohibited. It is hypothesized that such willingness was expressed by a significant proportion of never smokers who visited a California Indian casino in the year prior to the 2008 survey.

## Methods

### Sample of participants

The 2008 Adult California Tobacco Survey, a cross-sectional survey of tobacco use and behavior of California residents, utilized a two-stage sampling methodology similar to earlier versions of the survey [18]. The first stage of sampling entailed administration of a screener instrument by telephone to a sample of households (n = 22, 225) with at least one member over 17 years of age. From this first stage, all young adults between the ages of 18 and 29, all adult smokers, and a subset of adult non-smokers (based on racial/ethnic proportions) were selected for an extended interview about detailed smoking habits and behaviors, including perceptions and visits to casinos.

Data collection for the 2008 survey (n = 10, 397) was conducted between May 1, 2008 and February 22, 2009, and was approved by the Research Ethics Committee at the University of California, San Diego. Our secondary analysis of the data was exempt from review by the Institutional Review Board at the University of California, Irvine.

### Measures

**Figure 1:**
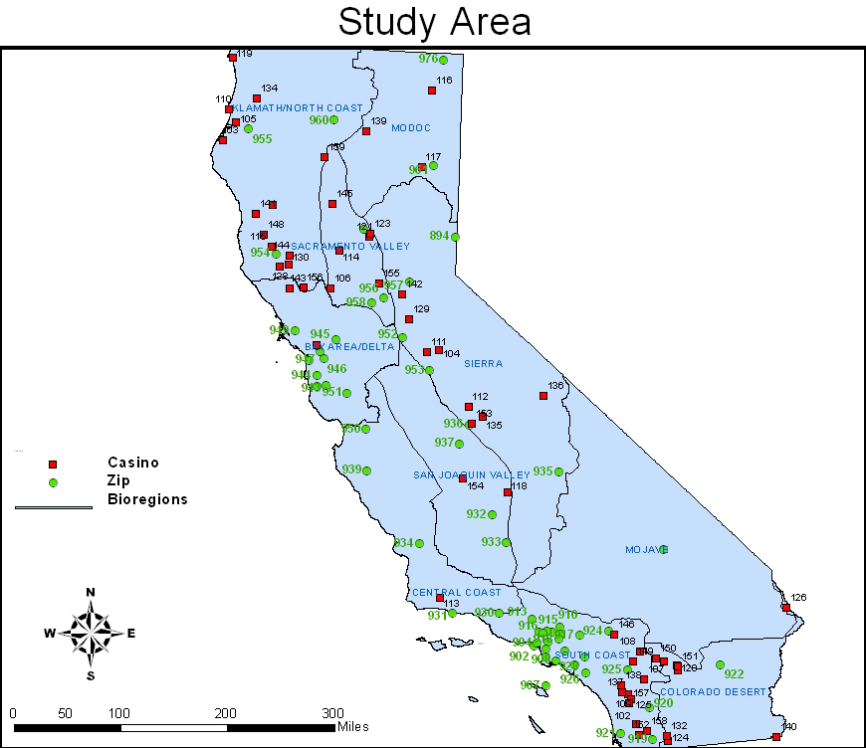
**Map of California illustrating distribution of participants' residence and location of 58 tribal casinos**.

The primary dependent variable was based on the question asked among all participants, "Have you visited an Indian casino in California in the past 12 months?". Predictors of this binary measure included sex, age, race/ethnicity, highest grade of school completed, smoking status and region of residence. The latter was constructed by aggregating 10 California bioregions by location, participant representation and casino density. The bioregion, based on the state's physiographic provinces [19], was an appropriate measure of geography because of the clustering of casinos (see Figure 1). The measure for smoking status included three groups, 1) never smokers who had never smoked 100 cigarettes or more in a lifetime, 2) former smokers who had smoked at least 100 cigarettes, but were not currently smoking, and 3) current smokers who had smoked at least 100 cigarettes. Categories of the remaining predictors of casino visitation are listed in Table 1.

**Table 1:** Odds of California residents having visited an Indian casino in the year prior to the 2008 survey

Measure	Patrons Column %	Non-Patrons Column %	Adjusted OR^c^ (95% C.I.)
Sample Size^a^	n = 3, 361	N = 7, 036	n = 10, 157^d^
**Demographics**			
*Sex*			
Male (vs. Female)	50.0%	49.2%	.96 (.79, 1.17)
*Age*			
18-29-year-olds	19.6%	21.2%	Referent
30-49-year-olds	37.1%	42.0%	1.00 (.78, 1.29)
≥ 50-year-olds	43.3%	36.8%	1.35 (1.11, 1.65) ^δ^
*Race/ethnicity*			
Non-Hispanic Caucasian	42.8%	48.2%	Referent
Non-Hispanic African-American	6.8%	5.5%	1.59 (1.29, 1.97) ^¥^
Hispanic	36.9%	30.9%	1.65 (1.24, 2.19) ^δ^
American Indian/Native Alaskan	3.0%	2.8%	.93 (.43, 2.02)
Asian/Pacific Islander/Other	10.5%	12.6%	1.27 (1.00, 1.60)*
*Highest grade of school completed*			
< 12^th ^grade	13.7%	14.4%	Referent
12^th ^grade	30.5%	23.3%	1.64 (1.03, 2.61)*
> 12^th ^grade	55.8%	62.3%	1.34 (.89, 2.03)
*California Residence (Bioregion)*			
Klamath/North Coast, Modoc, Col.^b^	11.1%	5.6%	Referent
Sacramento Valley and Sierra	18.4%	13.1%	.71 (.52, .98)*
San Francisco Bay Area/Delta	11.0%	20.9%	.28 (.19, .40) ^¥^
Central Coast and San Joaquin Val.	8.1%	8.7%	.47 (.30, .73) ^δ^
South Coast	51.4%	51.7%	.50 (.37, .67) ^¥^
**Smoking Status**			
Never Smoker	53.7%	66.7%	Referent
Former smoker	28.7%	22.9%	1.56 (1.22, 2.01) ^δ^
Current smoker	17.6%	10.4%	2.13 (1.77, 2.55) ^¥^

*p < .05; ^δ^*p *< .01; ^¥^*p *< .0001. ^a^Sample sizes are not weighted; percents are weighted. ^b^Referent for California residence includes Klamath/North Coast, Modoc, Colorado Desert and Mojave. ^c^Odds ratios are adjusted for all other variables in Table 1. ^d^Not equal to the sum of 3361 and 7036 due to missing data

A secondary analysis of casino patrons' perceptions and exposure to secondhand smoke was based on a series of questions asked only among those who visited a casino in the prior year (n = 3, 361). These included an ordinal measure of the amount of time spent around secondhand smoke during the last visit (i.e. no time at all - all of time), efforts to avoid secondhand smoke by moving around (i.e. changing card tables or moving to other slot machines), and willingness to extend or reduce one's stay if smoking were banned in the casino. Another hypothetical question, asked among all participants (n = 10, 397), inquired if a smoking ban would increase, decrease, or have no effect on the likeliness of visiting a California Indian casino.

### Approximating type of non-smoking section of last visited casino

Responses to measures of secondhand smoke in the Indian casinos were likely to vary according to the level of separation between the smoking and non-smoking sections. Thus, using the classification established by Jiang et al. (2011), all 58 California casinos in 2008 were categorized as either allowing smoking everywhere, or having a non-smoking section with no physical separation, semi-separation or complete physical separation from the smoking sections. Classification of 36 of the 58 casinos was provided by Ruo-Ting Jiang, PhD (personal comm.) who visually inspected the casinos; the remaining 22 casinos were assessed via telephone conversations with casino personnel, conducted independently by two trained research assistants.

The 2008 CTS did not query participants about which casino they last visited. Therefore, we approximated the type of non-smoking section of the last visited casino by two measures, 1) nearest casino to place of residence, and 2) probability of visiting a casino with complete versus incomplete physical separation (i.e. smoking allowed everywhere, no separation, or semi-separation). The probability was weighted by the number of casino slots, a surrogate for casino popularity, and the inverse proximity of the casino to place of residence (i.e. 1/distance in miles). The probability estimate included all casinos within 100 miles of residence, a value based on two considerations: 1) furthest distance between an individual's residence and the nearest casino was 92.1 miles, 2) average distance traveled to an Indian casino in Southern California was 64 miles based on a customer satisfaction survey [20] (*Note*: standard deviation not available). The probability (Pr_i, j_) was denoted by the equation: Pr_i, j _= ∑N_i, j _ × ((S_i, j_/D_i, j_)/∑(S_i, j_/D_i, j_)), where N_i, j _is equal to either 0 (incomplete physical separation) or 1 (complete physical separation) for the i^th ^casino of the j^th ^cluster of casinos within 100 miles of place of residence; D_i, j _represents distance in miles and S_i, j _represents number of slot machines. The addresses of casinos (N = 58) and centroids of the first three-digits of zip codes where study participants resided (n = 59) were geocoded using ArcGIS v10 [21]. Casino proximity was determined by calculating distance between the locations of the casinos and the centroid of a participant's three-digit zip code.

### Statistical analysis

The measures for smoking status and demographics were examined as predictors of a past-year casino visit in a logistic regression model, specified by the svy: logistic procedure in STATA v10 [22]. Given the probability sampling used in the 2008 CTS, 51 replicates of the original sample were generated for use by the jackknife method in obtaining unbiased variance estimates [23]. Given the sampling design, associations between categorical variables were tested using a second-order corrected *F *statistic [24]. However, the corrected *F *statistic was not employed in testing associations involving the approximated non-smoking section of the last visited casino; instead, a Pearson chi-square test was used.

## Results

### Smoking prevalence and predictors of casino visitation

In our study, the prevalence of current smoking was 17.6% for casino patrons and 10.4% for non-patrons (see Table 1). Adjusting for demographic variables, current smokers were approximately twice as likely to have visited an Indian casino compared to never smokers (OR = 2.13 (1.77, 2.55)). Significant predictors of casino visitation also included age, race/ethnicity, education, and region of residence. Participants aged 50 years and older were 35% more likely to have visited an Indian casino relative to young adults. Non-Hispanic African-Americans and Hispanics were approximately 60% more likely than Non-Hispanic Caucasians to have visited a casino in the prior year; similar findings were observed for participants with a high-school education versus those with less than a high-school education. Residence in the aggregated bioregions of Klamath/North Coast, Modoc, Colorado Desert and Mojave was highly predictive of casino visitation, a likely function of the high concentration of tribal casinos in Klamath/North Coast (15/58 casinos) and Colorado Desert (9/58 casinos). The spatial relationships and clustering of casinos in these sparsely populated regions of California are illustrated in Figure 1.

### Avoidance of secondhand smoke

**Figure 2:**
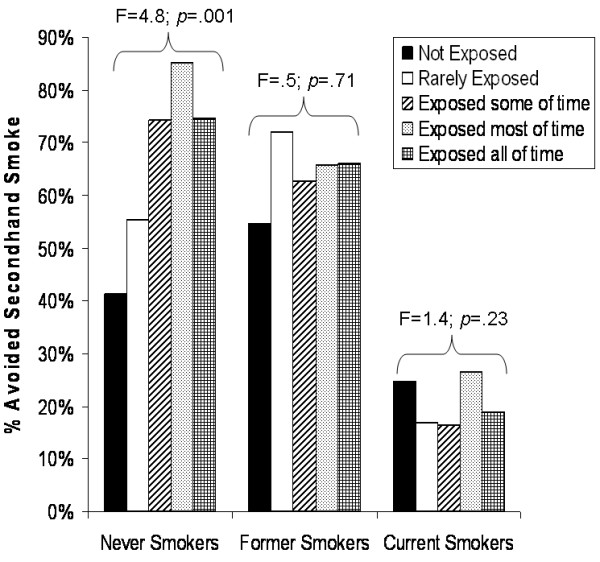
**Casino patrons' avoidance of secondhand smoke by reported levels of smoking and secondhand smoke exposure**.

A majority of casino patrons, 60.8%, attempted to avoid secondhand smoke by moving around the casino. This varied considerably by smoking status as reported by 71.8% of the never smokers, 64.5% of the former smokers, and 20.4% of current smokers (F(1.6, 79.1) = 66.8, *p *< .0001). Only among the never smokers did avoidance of secondhand smoke vary significantly by degree of secondhand smoke exposure in the casino (F(3.7, 185.6) = 4.8, *p *= .001) (refer to Figure 2). In contrast, significant associations were neither observed for the former smokers (F(3.5, 175.8) = .5, *p *= .71) nor the current smokers (F(3.8, 192.4) = 1.4, *p *= .23). Participants who reported little or no secondhand smoke exposure and also reported efforts to avoid such exposure may have moved successfully to a non-smoking section.

**Figure 3:**
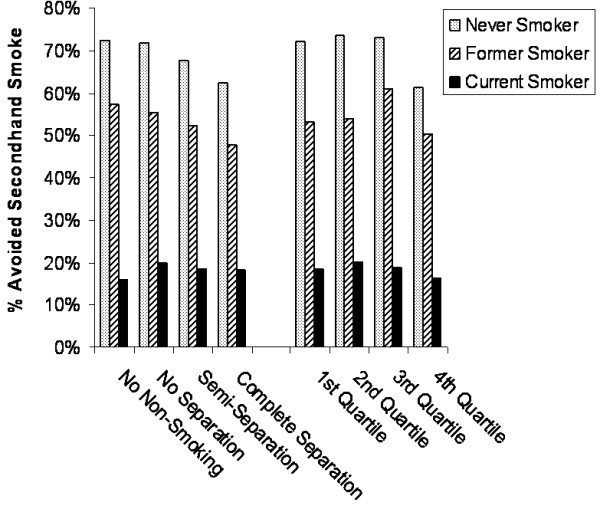
**Avoidance of secondhand smoke by the nearest casino's non-smoking section and probability (quartile) of visiting a casino with an enclosed non-smoking section**.

Among the 58 California casinos, 21 were identified as being closest in proximity in miles to the centroids of zip codes where past-year patrons resided; the median distance was 36.1 miles. Three of the 59 centroids had only a single casino within a 100 mile distance, whereas, five centroids had 19 casinos within a 100 mile distance. As illustrated in Figure 3, a negative association, although not statistically significant (*χ*_(3 d.f.)_^2 ^= 5.8, *p *= .12), was observed between never-smokers' avoidance of secondhand smoke and their nearest casinos' type of non-smoking section. This trend was apparent neither for the former nor current smokers.

Accounting for casino distance and popularity (i.e. no. of slots), the probability of visiting a casino with complete physical separation between smoking and non-smoking sections was highly associated with avoidance of secondhand smoke among the never smokers (*χ*_(3 d.f.)_^2 ^= 12.3, *p *= .006). For these individuals, minimal variation in avoidance of secondhand smoke was observed across the first three probability quartiles: Quartile one (72.2%), quartile two (73.6%), quartile three (73.1%) (Refer to Figure 3; quartiles were illustrated due to the skewed probability distribution). In contrast, 61.3% of never smokers from the fourth quartile (range of probabilities: .366 - 1.0) avoided secondhand smoke. Unlike the never smokers, there was no significant association between the probability quartiles and avoidance of secondhand smoke for the former or current smokers.

### Views about a hypothetical smoking ban

A total of 42.7% of casino patrons indicated that they would extend their stay if smoking were prohibited; another 48.8% reported that their stay would not be affected by such a ban; and the remaining 8.5%, predominately smokers, reported a shortened stay if there were such a ban. In a separate question asked among all participants, 24.3% expressed a greater interest in visiting a casino if smoking were prohibited, 6.3% expressed a diminished interest, and 69.4% expressed indifference. Though, the majority of participants in the 2008 CTS (67.2%) indicated their support for a smoking ban in California Indian casinos.

As illustrated in Table 2, attitudes regarding a smoking ban varied considerably by smoking status. Almost 50% of the never smokers and former smokers who visited a casino in the prior year reported a willingness to extend their stay if a ban were implemented; only 13% of current smokers expressed such sentiment (F(3.1, 154.9) = 38.9, *p *< .0001). A similar finding was observed among the casino patrons (F(3.0, 151.0) = 37.9, *p *< .0001) and non-patrons (F(2.8, 139.8) = 13.7, *p *< .0001) when asked about their willingness to visit an Indian casino if smoking were banned. Compared to the patrons, the non-patrons expressed a greater indifference to a hypothetical smoking ban.

**Table 2:** Willingness to have stayed and visited a California Indian casino if smoking were prohibited

Measure	Never smokers (63.2%)	Former smokers (24.5%)	Current smokers (12.3%)	F-Test^c^
**Patrons (n = 3361)**				
*Casino Stay *^a^	Column %	Column %	Column %	
Shorter length of stay	4.4%	3.7%	29.3%	
Same length of stay	45.9%	49.0%	57.5%	
Longer length of stay	49.7%	47.3%	13.2%	38.9^¥^
*Casino Visit *^b^				
Visit less likely	2.6%	2.0%	22.5%	
No difference	55.3%	62.2%	70.5%	
Visit more likely	42.1%	35.8%	7.0%	37.9^¥^
**Non-Patrons (n = 7015)**				
*Casino Visit *^b^	Column %	Column %	Column %	
Visit less likely	5.7%	6.7%	11.2%	
No difference	70.6%	75.0%	83.3%	
Visit more likely	23.7%	18.3%	5.5%	13.7^¥^

^¥^*p *< .0001. ^a^Hypothetical question about shortening or extending one's stay, asked only among past-year patrons. ^b^Hypothetical question about visiting a casino in the future, asked among patrons and non-patrons. ^c^F statistic (svy: tab) is based on a second order correction

## Discussion

The prevalence of cigarette smoking was considerably higher in casino patrons (17.6%) compared to non-patrons (10.4%), but, not nearly as high as the 50% estimate reported by gaming lobbyists [25]. Our finding was more consistent with the estimates of smoking among gamblers at casinos in Las Vegas, NV (20.3%) and Reno/Sparks, NV (21.5%), taking into account the difference in smoking prevalence between California and Nevada [26]. Though, unlike the Nevada gamblers, the smoking prevalence among California Indian casino patrons appreciably exceeded the state prevalence (17.6% vs. 12.3%, respectively). This may be attributed to a variety of methodological differences between our study and the Nevada study, ranging from sampling to the actual prevalence of smoking in the respective tourist destinations. Casinos in Nevada attract national and international tourists whom exhibit smoking rates comparable to their places of residence.

While much of the debate over smoking bans in casinos has centered on smokers, the crux of the debate is non-smokers' exposure and perceptions of secondhand smoke. This is particularly evident in California where the majority of Indian casino patrons and non-patrons do not smoke. Similar to the smoking bans in restaurants and bars in California, a smoking ban in tribal casinos is unlikely to affect casino businesses negatively [11,12]. Given the majority of non-smokers in the sample (87.7%), there were more patrons and non-patrons who expressed a greater versus lesser desire to visit an Indian casino if smoking were banned. The significant association between exposure to and avoidance of secondhand smoke, observed only among the never smokers, highlights the importance of providing a smoke-free environment for patrons. More importantly, there is a pressing need to eliminate secondhand smoke exposure among employees of casinos. One survey indicated that a majority of London casino workers (71%) were exposed to high levels of secondhand smoke, and a majority (65%) preferred a smoke-free environment [27]. Ventilation systems can reduce the odor and haze associated with secondhand smoke, but, according to the American Society of Heating, Refrigeration, and Air-Conditioning Engineers [28], they cannot reduce health-related risks; thus, they are not a viable alternative to smoke-free policies. Similarly, enclosed non-smoking sections are not recommended despite our observation that the never smokers exhibited less avoidance of secondhand smoke in casinos with such facilities. Advocacy for the enclosed non-smoking section would not resolve the occupational hazards associated with exposure to secondhand smoke in other parts of the casino.

### Study strengths and limitations

Analysis of the 2008 California Tobacco Survey provided one of the few opportunities to estimate the prevalence of smoking status and exposure to secondhand smoke among patrons of California Indian casinos. Yet, its use came with limitations. The primary weakness of this study was the lack of information on the last visited casino and residential addresses of participants; thus, a crude estimation was based on number of slot machines and proximity of a casino to a participant's geocoded centroid. Further, most study participants were sampled in regions in California where casinos are sparse, an additional complication to our estimation. However, our assertion that proximity was a prime determinant of the last visited casino was warranted, given the significant association between casino visitation and residence in a casino-populated region (i.e. Klamath/North Coast, Modoc, etc.). Our study was also hampered by a use of a cross-sectional design; use of different methods in ascertaining a casino's non-smoking section (i.e. visit vs. telephone call); lack of data on actual smoking in a casino; and location where exposure to smoke occurred (e.g., slot machines vs. card tables). Despite the study limitations, a significant association was observed between type of non-smoking section and avoidance of secondhand smoke. The magnitude of this association was likely underestimated due to non-differential misclassification (i.e. measurement error) of the non-smoking sections of casinos.

## Conclusions

The findings of this study have important implications for the support of a smoking ban in tribal casinos in California. The data indicates that such a ban would increase casino visitation, possibly resulting in greater revenue and greater customer satisfaction. At the moment, the public health community should continue to support initiatives such as California's Clean Air Project, whose mission is to provide technical assistance for tribal nations' voluntary adoption of smoke-free policies.

## Competing interests

The authors declare that they have no competing interests.

## Authors' contributions

DST conceived the study design, conducted most of the analyses, and drafted the manuscript. 

JW developed the probability calculation for the last visited casino, geocoded casinos and participants' residence (i.e. centroid), and contributed to the methods section.

WAD conducted the California Tobacco Survey that generated the data for this study, provided methodological input on design and measurements, and edited various sections of the manuscript. All authors read and approved the final manuscript.

## Pre-publication history

The pre-publication history for this paper can be accessed here:

http://www.biomedcentral.com/1471-2458/12/144/prepub

## References

[B1] Bal DG Kizer KW Felten PG Mozar HN Niemeyer D Reducing tobacco consumption in California. Development of a statewide anti-tobacco use campaign JAMA 1990 264 12 1570 1574 10.1001/jama.1990.03450120082034 2395199

[B2] Francis JA Abramsohn EM Park HY Policy-driven tobacco control Tob Control 2010 19 Suppl 1 i16 i20 20382645 10.1136/tc.2009.030718 PMC2976507

[B3] Glantz SA Smith LR The effect of ordinances requiring smoke-free restaurants on restaurant sales Am J Public Health 1994 84 7 1081 1085 10.2105/AJPH.84.7.1081 8017529 PMC1614757

[B4] Stolzenberg L D'Alessio SJ Is nonsmoking dangerous to the health of restaurants? The effect of California's indoor smoking ban on restaurant revenues Eval Rev 2007 31 1 75 92 10.1177/0193841X06284814 17259576

[B5] Centers for Disease Control and Prevention Tobacco use among adults-United States, 2005 MMWR Morb Mortal Wkly Rep 2006 55 42 1145 1148 17065979

[B6] Harper T Smoking and gambling: a trance inducing ritual Tob Control 2003 12 2 231 233 10.1136/tc.12.2.231 12773737 PMC1747713

[B7] Petry NM Oncken C Cigarette smoking is associated with increased severity of gambling problems in treatment-seeking gamblers Addiction 2002 97 6 745 753 10.1046/j.1360-0443.2002.00163.x 12084144

[B8] Grant BF Hasin DS Chou SP Stinson FS Dawson DA Nicotine dependence and psychiatric disorders in the United States: results from the national epidemiologic survey on alcohol and related conditions Arch Gen Psychiatry 2004 61 11 1107 1115 10.1001/archpsyc.61.11.1107 15520358

[B9] Mandel LL Glantz SA Hedging their bets: tobacco and gambling industries work against smoke-free policies Tob Control 2004 13 3 268 276 10.1136/tc.2004.007484 15333883 PMC1747893

[B10] Lal A Siahpush M The effect of smoke-free policies on electronic gaming machine expenditure in Victoria, Australia J Epidemiol Community Health 2008 62 1 11 15 10.1136/jech.2006.051557 18079327

[B11] Glantz SA Wilson-Loots R No association of smoke-free ordinances with profits from bingo and charitable games in Massachusetts Tob Control 2003 12 4 411 413 10.1136/tc.12.4.411 14660778 PMC1747798

[B12] Mandel LL Alamar BC Glantz SA Smoke-free law did not affect revenue from gaming in Delaware Tob Control 2005 14 1 10 12 10.1136/tc.2004.008755 15735294 PMC1747973

[B13] Jiang RT Cheng KC Acevedo-Bolton V Klepeis NE Repace JL Ott WR Hildemann LM Measurement of fine particles and smoking activity in a statewide survey of 36 California Indian casinos J Expo Sci Environ Epidemiol 2011 21 1 31 41 10.1038/jes.2009.75 20160761 PMC3007589

[B14] World Health Organization The WHO Framework Convention on Tobacco Control 2003 Geneva, Switzerland: WHO Framework Convention on Tobacco Control

[B15] Erazo M Iglesias V Droppelmann A Acuna M Peruga A Breysse PN Navas-Acien A Secondhand tobacco smoke in bars and restaurants in Santiago, Chile: evaluation of partial smoking ban legislation in public places Tob Control 2010 19 6 469 474 10.1136/tc.2009.035402 20798021 PMC2991072

[B16] Sebrie EM Glantz SA "Accommodating" smoke-free policies: tobacco industry's Courtesy of Choice programme in Latin America Tob Control 2007 16 5 e6 10.1136/tc.2006.018275 17897975 PMC2598557

[B17] Dearlove JV Bialous SA Glantz SA Tobacco industry manipulation of the hospitality industry to maintain smoking in public places Tob Control 2002 11 2 94 104 10.1136/tc.11.2.94 12034999 PMC1763854

[B18] Al-Delaimy WK Edland S Pierce JP Mills AL White MM Technical Report on Analytical Methods and Approaches Used in the 2008 California Tobacco Survey Analysis. Vol 1: Data Collection Methodology, Public Use Data File Documentation, Individual Item Responses 2009 La Jolla: University California, San Diego

[B19] California Biodiversity Council Bioregions of California http://biodiversity.ca.gov/Bioregions/INACC.pdf

[B20] JD Power Report Socal majority wants smoke-free Indian casinos http://500nations.com/news/California/20080707.asp

[B21] ArcGISv10 Redlands: ESRI

[B22] StataCorp Stata statistical software 2010 9.0 College Station, TX: Stata Corporation

[B23] Al-Delaimy WK Edland S Pierce JP White MM Technical Report on Analytical Methods and Approaches Used in the 2008 California Tobacco Survey Analysis. Vol 2: Statistical Methodology, Public Use Data File Documentation, Individual Item Responses 2009 La Jolla: University California, San Diego

[B24] Rao JNK Thomas DR *Analysis of Complex Surveys*. *Chi-squared tests for contingency tables* 1989 New York: Wiley

[B25] Dobra J Economic impacts of the proposed OSHA smoking ban on the state of Nevada Philip Morris 1996 2072360688/2072360711

[B26] Pritsos CA Pritsos KL Spears KE Smoking rates among gamblers at Nevada casinos mirror US smoking rate Tob Control 2008 17 2 82 85 10.1136/tc.2007.021196 18276735

[B27] Pilkington PA Gray S Gilmore AB Daykin N Attitudes towards second hand smoke amongst a highly exposed workforce: survey of London casino workers J Public Health (Oxf) 2006 28 2 104 110 10.1093/pubmed/fdi086 16497788

[B28] Samet J Bohanon HR Coultas DB Houston TP Persily AK Schoen LJ Spengler J Callaway CA ASHRAE position document on environmental tobacco smoke American Society of Heating, Refrigerating and Air-Conditioning Engineers 2005

